# Supplementary material for: Does the urban low-carbon transition promote the residents’ health consumption?
Source: PLoS One. 2025 Mar 21;20(3):e0319835. doi: 10.1371/journal.pone.0319835 (PMC11928064; doi:10.1371/journal.pone.0319835)
Supplement: Attachment — Submitted filename: Comments.docx [file pone.0319835.s002.docx]

This paper uses fixed effects models to discuss the impact of low-carbon transformation on residents' health consumption and explores the mechanisms from the perspectives of residents' health awareness and urban environmental quality. Although the paper has conducted endogeneity analysis and a series of robustness tests, there are still some issues that need further explanation and revision. The specific opinions are as follows:

1. In terms of marginal contribution: Since there has already been research focusing on the impact of low-carbon transition on residents’ health or consumption, the authors should emphasize why it is important to focus on residents’ health consumption within the context of low-carbon transition research. The article should clarify what questions it addresses, what research perspective it adds to the existing literature, its practical significance, and its theoretical implications. Furthermore, the authors mention that this article establishes a theoretical analysis framework to examine the impact of low-carbon transition on residents’ health consumption. However, the theoretical analysis framework presented in the following sections is based on existing literature and does not offer significant theoretical contributions. Therefore, the authors need to reconsider whether the contributions in this section are appropriate.

2. The logic of the introduction is somewhat tenuous, especially in the second paragraph. The author first mentions the reasons for implementing a low-carbon transition in the second paragraph, then abruptly shifts the topic to the current economic situation and introduces the importance of consumption. It is difficult to emphasize why the low-carbon transition should prioritize the importance of residents' health.

3. The micro data of this paper comes from CGSS database in 2018 and 2021. As far as I know, CGSS data are collected in many periods, so why the author only uses the data of these two periods, such as the data of previous years can also be used? If not, it should be specified in the article.

4. In terms of variable definition, the article defines health consumption based on the purchase of four types of insurance. This method of defining variables is skeptical, and the author should clarify whether any existing literature has adopted this approach. Among the four types of insurance are health insurance and pension insurance; however, the relationship between pension insurance and residents’ health consumption is not closely related. Additionally, the author assigns values based on the number of insurance purchases, which fails to accurately reflect the differences in health consumption among residents. This is because basic health insurance is a social welfare benefit that residents are entitled to. If residents purchase commercial health insurance, their premium expenditures and level of concern for health are often higher, but the variable definition used in the article does not accurately capture this. Existing research on residents’ health consumption typically uses their expenditure on medical and health services as a research variable. If there are relevant variables in the data used in this article, it is recommended that the author include them in the robustness check section to enhance the reliability of the research conclusions.

5. In terms of model specification, both Model 1 and Model 2 should account for clustered robust standard errors at the city level to avoid bias in the baseline regression results due to the correlation among individuals within the same city.

6. Mechanism Analysis Section: (1) The definition of the mechanism variable “residents’ health awareness” in this article may not be entirely appropriate. The article is based on the questionnaire questions “In the past four weeks, how often has your work or other daily activities been affected by health problems? Always, often, sometimes = 1; other = 0” to define residents’ health awareness, a potential issue with the questionnaire is that the questions may reflect more on the extent to which residents are affected by health issues in their work and daily lives, rather than their concern for their own health problems. (2) Unlike existing literature that uses urban air quality as a representation of urban environmental quality, this article’s choice of urban green space area as a proxy variable for urban environmental quality may be incomplete. It is recommended that the authors appropriately include air quality for examination in this section.

7. Robustness Check Section: (1) Based on the research subjects and scenarios of this paper, using a fixed effects model for estimation is more appropriate. However, the use of a random effects model in this section as a robustness check does not resolve the issues present in the baseline estimation results; rather, the results obtained from the random effects model are coarser than those from the fixed effects model. Additionally, there is a problem in the presentation of the estimation results in this section; according to the estimation results in Table 3, Column 1, it should be a 90% confidence interval, not a 95% confidence interval. (2) The author should not only apply winsorization to income but should also apply it to all continuous variables in the control variables to mitigate the bias caused by outliers on the baseline regression results. (3) The author should consider adding more robustness checks, such as replacing the dependent or independent variables, or changing the level of fixed effects or so on, to enhance the credibility of the baseline regression results.

8. Endogeneity Analysis Section: To address potential endogeneity issues in this article, the author uses the urban green space coverage rate from 2006 as an instrumental variable for estimation. However, a possible problem is that this instrumental variable may not satisfy the exclusion restriction assumption, as the urban green space coverage rate can directly affect residents’ health rather than only indirectly influence residents’ health through its impact on urban low-carbon transformation. Therefore, it does not meet the exclusion condition. The author should include relevant tests for the exogeneity and exclusion restriction of the instrumental variable to assess its reliability.
