# Supplementary material for: Does the urban low-carbon transition promote the residents’ health consumption?
Source: PLoS One. 2025 Mar 21;20(3):e0319835. doi: 10.1371/journal.pone.0319835 (PMC11928064; doi:10.1371/journal.pone.0319835)
Supplement: Attachment — Submitted filename: Response to reviewers.docx [file pone.0319835.s004.docx]

**Responses to editor of PONE: regarding the paper** “Does the urban low-carbon transition promote the residents’ health consumption?” (Ref.: PONE-D-24-39039)

By

Pian Chen, Yanchi Chen

Dear Editor:

First of all, we would like to thank the reviewers for their serious and thorough work in refereeing this paper. All the reviewers’ suggestions have now been incorporated into the paper for revisions. A detailed description of the revisions undertaken is given below.

We think the reviewers’ queries and suggestions have been adequately addressed, and hope that the paper is now suitable for publication in PLOS ONE.

Thank you very much. We look forward to hearing from you.

Yours sincerely,

Corresponding author

Dr. Pian Chen

Institute of Food and Strategic Reserves

Nanjing University of Finance and Economics

Nanjing, 210023, China

**Point-by-point responses to the reviewers’ comments on the manuscript**

“Does the urban low-carbon transition promote the residents’ health consumption?” (Ref.: PONE-D-24-39039)

By

Pian Chen, Yanchi Chen

The authors would like to thank the Reviewers for their careful review of the manuscript and providing insightful comments and suggestions. The following responses have been prepared to address the reviewers’ comments. All the comments have been addressed in the revised manuscript (highlighted in red).

=============================================================**Reviewer #1**

***Abstract***

***Comments 1****. The abstract follows a logical structure and provides a good overview of the study by summarizing the purpose, methodology, results, and conclusions. However, unfortunately, I did not see specific statistical results. Providing key results could have strengthened the impact of the study findings.*

**Response:** Thanks for your useful suggestions. We have added key statistical results to the abstract as suggested by the reviewer.

Please see Lines 25-26 in the revised manuscript.

***Comments 2****. should have added the policy recommendations of the study, etc. after the conclusions of the study.*

**Response:** Thanks for your useful suggestions. We have added relevant policy recommendations to the summary as suggested by the reviewer.

Please see Lines 34-38 in the revised manuscript.

**Presentation**

***Comments 1****. line 40, progressive growth in health needs. Providing relevant data would better visualize this.*

**Response:** Thanks for your useful suggestions. We have added data related to the growth of global health consumption as suggested by the reviewer.

Please see Lines 45-48 in the revised manuscript.

***Comments 2****. Lack of a clear definition of “urban low carbon”.*

**Response:** Thanks for your useful suggestions. We have added a definition of “urban low-carbon transition” as suggested by the reviewer.

Please see Lines 60-63 in the revised manuscript.

**Literature Review and Theoretical Analysis**

***Comments 1****. The literature review analyzes the current state of research in a comprehensive way, but your argument is mainly based on Chinese scholars and not on research in other countries.*

**Response:** Thanks for your useful suggestions. We have added relevant studies by foreign scholars in the literature review section.

Please see Lines 100-108, 116-122 in the revised manuscript.

***Comments 2****. In your theoretical analysis, it would be more comprehensive if you add more specific theories or formulaic derivations in the supply and demand analysis.*

**Response:** Thanks for your useful suggestions. We have added the relevant theory to the supply side and demand side analysis respectively.

Please see Lines 149-211 in the revised manuscript.

**Analysis of mechanisms**

***Comments 1****. For your explanatory variables, you have used the question “Are you currently enrolled in any of the following social security programs? Urban basic medical insurance/new rural cooperative medical insurance/public medical care cooperative medical insurance/public medical care; urban/rural basic pension insurance; commercial medical insurance; commercial pension insurance. insurance.” is measured by responses to a questionnaire. But this does not reflect the full picture of consumer spending, what is your rationale for measuring it this way?*

**Response:** Scholars have delineated “health consumption” from various viewpoints, and this paper contends that health consumption encompasses activities aimed at maintaining, enhancing, or restoring health. This includes, but is not confined to, the acquisition of medical services, nutritious food, health insurance, etc., and can be classified into active and passive health consumption. Active health consumption highlights the proactive choices and behaviors of individual consumers, such as investing in health insurance. Conversely, passive health consumption is contingent upon external events, such as the onset of diseases. In contrast to passive health consumption, active health consumption more accurately represents people’ willingness and capacity to engage in health-related expenditures. Furthermore, while pension insurance does not directly offer health services or products, it indirectly promotes an individual’s health by guaranteeing financial stability. A stable income enables individuals to invest more liberally in their health, including access to healthcare services. This paper utilizes the research of Mocan et al. (2003) and integrates the CGSS questionnaire to quantify health consumption through the expenditure on social security services. An increase in the acquisition of social security programs by inhabitants correlates with a heightened degree of health consumption, and conversely.

**Analysis of empirical results**

***Comments 1****. In your section on heterogeneity analysis, you suggest comparing the findings of other scholars to increase credibility.*

**Response:** Thanks for your useful suggestions. We have added corresponding existing research results to the analysis of income heterogeneity, gender heterogeneity, and heterogeneity in the level of urban development, respectively.

Please see Lines 401-402, 423-424, 443-444 in the revised manuscript.

**Conclusion and Policy Implications**

***Comments 1****. The discussion section is too brief and does not go into depth from the findings. It should be integrated with the existing literature and suggest policy implications at the government level.*

**Response:** Thanks for your useful suggestions. We have made relevant revisions to the conclusion section based on the reviewers’ suggestions and have made relevant policy recommendations at the government level based on the conclusion.

Please see Lines 466-468,481-520 in the revised manuscript.

**Reviewer #2:**

***Comments 1.*** *In terms of marginal contribution: Since there has already been research focusing on the impact of low-carbon transition on residents’ health or consumption, the authors should emphasize why it is important to focus on residents’ health consumption within the context of low-carbon transition research. The article should clarify what questions it addresses, what research perspective it adds to the existing literature, its practical significance, and its theoretical implications. Furthermore, the authors mention that this article establishes a theoretical analysis framework to examine the impact of low-carbon transition on residents’ health consumption. However, the theoretical analysis framework presented in the following sections is based on existing literature and does not offer significant theoretical contributions. Therefore, the authors need to reconsider whether the contributions in this section are appropriate.*

**Response:** Thanks for your useful suggestions. We have reworked the theoretical contributions section based on reviewer suggestions.

Please see Lines 71-86 in the revised manuscript.

***Comments 2.*** *The logic of the introduction is somewhat tenuous, especially in the second paragraph. The author first mentions the reasons for implementing a low-carbon transition in the second paragraph, then abruptly shifts the topic to the current economic situation and introduces the importance of consumption. It is difficult to emphasize why the low-carbon transition should prioritize the importance of residents’ health.*

**Response:** Thanks for your useful suggestions. We have reworked the second part of the introduction to make it more logical for the article, as suggested by the reviewer.

Please see Lines 56-70 in the revised manuscript.

***Comments 3.*** *The micro data of this paper comes from CGSS database in 2018 and 2021. As far as I know, CGSS data are collected in many periods, so why the author only uses the data of these two periods, such as the data of previous years can also be used? If not, it should be specified in the article.*

**Response:** Thanks for your useful suggestions. We have added instructions for using data from these two issues to the article as suggested by the reviewer.

Please see Lines 298-304 in the revised manuscript.

***Comments 4.*** *In terms of variable definition, the article defines health consumption based on the purchase of four types of insurance. This method of defining variables is skeptical, and the author should clarify whether any existing literature has adopted this approach. Among the four types of insurance are health insurance and pension insurance; however, the relationship between pension insurance and residents’ health consumption is not closely related. Additionally, the author assigns values based on the number of insurance purchases, which fails to accurately reflect the differences in health consumption among residents. This is because basic health insurance is a social welfare benefit that residents are entitled to. If residents purchase commercial health insurance, their premium expenditures and level of concern for health are often higher, but the variable definition used in the article does not accurately capture this. Existing research on residents’ health consumption typically uses their expenditure on medical and health services as a research variable. If there are relevant variables in the data used in this article, it is recommended that the author include them in the robustness check section to enhance the reliability of the research conclusions.*

**Response:** Thanks for your useful suggestions. Scholars have delineated "health consumption" from various viewpoints, and this paper contends that health consumption encompasses activities aimed at maintaining, enhancing, or restoring health. This includes, but is not confined to, the acquisition of medical services, nutritious food, health insurance, etc., and can be classified into active and passive health consumption. Active health consumption highlights the proactive choices and behaviors of individual consumers, such as investing in health insurance. Conversely, passive health consumption is contingent upon external events, such as the onset of diseases. In contrast to passive health consumption, active health consumption more accurately represents people’ willingness and capacity to engage in health-related expenditures. Furthermore, while pension insurance does not directly offer health services or products, it indirectly promotes an individual’s health by guaranteeing financial stability. A stable income enables individuals to invest more liberally in their health, including access to healthcare services. This paper utilizes the research of Mocan et al. (2003) and integrates the CGSS questionnaire to quantify health consumption through the expenditure on social security services. An increase in the acquisition of social security programs by inhabitants correlates with a heightened degree of health consumption, and conversely.

Furthermore, the database’s exclusion of residents’ healthcare service expenditures precludes the possibility of conducting the essential robustness test. We trust you will comprehend that this paper incorporates the decision to get commercial medical insurance as a robustness test for residents’ health consumption variables.

Please see Lines 324-337, 338-339(Table 3) in the revised manuscript.

***Comments 5.*** *In terms of model specification, both Model 1 and Model 2 should account for clustered robust standard errors at the city level to avoid bias in the baseline regression results due to the correlation among individuals within the same city.*

**Response:** Thanks for your useful suggestions. We have modified the treatment of standard errors to enhance their suitability for cluster analysis at the city level.

Please see Lines 257, 321-322(Table 2), 338-339(Table 3), 356-358(Table 4), 390-391(Table 5), 455-456(Table 6) in the revised manuscript.

***Comments 6.*** *Mechanism Analysis Section: (1) The definition of the mechanism variable “residents’ health awareness” in this article may not be entirely appropriate. The article is based on the questionnaire questions “In the past four weeks, how often has your work or other daily activities been affected by health problems? Always, often, sometimes = 1; other = 0” to define residents’ health awareness, a potential issue with the questionnaire is that the questions may reflect more on the extent to which residents are affected by health issues in their work and daily lives, rather than their concern for their own health problems. (2) Unlike existing literature that uses urban air quality as a representation of urban environmental quality, this article’s choice of urban green space area as a proxy variable for urban environmental quality may be incomplete. It is recommended that the authors appropriately include air quality for examination in this section.*

**Response:** Thanks for your useful suggestions. (1) Rationale for Assessing Residents’ Health Awareness: This research contends that analyzing the prevalence of health issues encountered by residents in their work or everyday activities can operate as an indirect indicator of their health awareness level. When residents have recurrent health issues that disrupt their employment or everyday activities, such experiences may act as a catalyst for heightened health awareness and encourage individuals to adopt a more proactive approach to health management. Conversely, if inhabitants consider themselves physically strong and well, they may exhibit diminished vigilance and investment in health maintenance, perhaps resulting in a proportionate decline in health awareness. In summary, a probable negative association exists between the prevalence of health issues and the level of individual health awareness; specifically, a growth in health problems is likely inversely connected to the improvement of health awareness. (2) This research primarily posits that urban low-carbon transition influences residents’ health consumption via enhancing the level of habitat environment rather than urban environmental quality, following a literature analysis and reflection. Consequently, according to Zhou’s (2024) research, habitat environment level is assessed by the extent of urban green space. The theoretical analysis and mechanism test have been amended. (References: Zhou, J. Urban ecological human settlements design based on green and low-carbon concept. Journal of Computational Methods in Sciences and Engineering.2024, 24(1), 303-309. DOI: 10.3233/JCM-237049)

Please see Lines 232-247, 376-389, 390-391(Table 5) in the revised manuscript.

***Comments 7.*** *Robustness Check Section: (1) Based on the research subjects and scenarios of this paper, using a fixed effects model for estimation is more appropriate. However, the use of a random effects model in this section as a robustness check does not resolve the issues present in the baseline estimation results; rather, the results obtained from the random effects model are coarser than those from the fixed effects model. Additionally, there is a problem in the presentation of the estimation results in this section; according to the estimation results in Table 3, Column 1, it should be a 90% confidence interval, not a 95% confidence interval. (2) The author should not only apply winsorization to income but should also apply it to all continuous variables in the control variables to mitigate the bias caused by outliers on the baseline regression results. (3) The author should consider adding more robustness checks, such as replacing the dependent or independent variables, or changing the level of fixed effects or so on, to enhance the credibility of the baseline regression results.*

**Response:** Thanks for your useful suggestions. (1) As suggested by the reviewers, we removed the tests related to the use of random effects models in the robustness testing section. (2) The correlation test incorporating the substitution of explanatory factors was introduced. Robustness tests were performed to evaluate the decision to obtain commercial health insurance as a measure of home health consumption. Furthermore, we standardize all continuous control variables prior to performing the robustness test.

Please see Lines 324-337, 338-339(Table 3) in the revised manuscript.

**Comments 8.** Endogeneity Analysis Section: To address potential endogeneity issues in this article, the author uses the urban green space coverage rate from 2006 as an instrumental variable for estimation. However, a possible problem is that this instrumental variable may not satisfy the exclusion restriction assumption, as the urban green space coverage rate can directly affect residents’ health rather than only indirectly influence residents’ health through its impact on urban low-carbon transformation. Therefore, it does not meet the exclusion condition. The author should include relevant tests for the exogeneity and exclusion restriction of the instrumental variable to assess its reliability.

**Response:** Thanks for your useful suggestions. We conduct the endogeneity test again as recommended by the reviewers. According to Fu et al. (2021), inverse temperature served as an instrumental variable for estimate. (References: Fu, S., Viard, V. B., & Zhang, P. Air pollution and manufacturing firm productivity: Nationwide estimates for China. The Economic Journal.2021, 131(640), 3241-3273.)

Please see Lines 341-355, 356-358(Table 4) in the revised manuscript.

**Reviewer #3:**

***Comments 1.*** *The contribution in the Introduction section is not sufficiently clear. Please reorganize this section and explicitly articulate the novelty of your paper in comparison to existing literature.*

**Response:** Thanks for your useful suggestions. We have reworked the theoretical contributions section based on reviewer suggestion.

Please see Lines 71-86 in the revised manuscript.

***Comments 2.*** *Please clarify whether calculating TFP at the city level can effectively capture industry heterogeneity within the city. Service and tourism industries may not experience the same degree of carbon reduction in their transformation.*

**Response:** Thanks for your useful suggestions. We think that city-level total factor productivity (TFP) calculations can somewhat capture industry variation within cities, but they may also have certain drawbacks. TFP can somewhat reflect the variations in productivity between industries within a city because it takes into account variables like management effectiveness and technology advancement, which fluctuate across industries. Total factor productivity, on the other hand, is a broad metric that might not adequately represent the minute variations among industries. TFP might not fairly represent industry heterogeneity, especially when it is primarily represented by certain characteristics like energy use efficiency and carbon emission intensity. As a significant component of the urban economy, the service and tourist sectors, for instance, could not have experienced the same level of carbon emission reduction during the shift. In order to somewhat offset the restrictions, this article incorporates carbon emission indicators into the measurement of urban total factor production.

***Comments 3.*** *Please specify whether the empirical results are based on clustered standard errors.*

**Response:** Thanks for your useful suggestions. We have modified the treatment of standard errors to enhance their suitability for cluster analysis at the city level.

Please see Lines 257, 321-322(Table 2), 338-339(Table 3), 356-358(Table 4), 390-391(Table 5), 455-456(Table 6) in the revised manuscript.

***Comments 4.*** *The F-statistic for the weak instrument is high, and the coefficient of IV is similar to OLS. Please discuss whether the instrument satisfies the exogeneity assumption.*

**Response:** Thanks for your useful suggestions. We conduct the endogeneity test again as recommended by the reviewers. According to Fu et al. (2021), inverse temperature served as an instrumental variable for estimate. (References: Fu, S., Viard, V. B., & Zhang, P. Air pollution and manufacturing firm productivity: Nationwide estimates for China. The Economic Journal.2021, 131(640), 3241-3273.)

https://doi.org/10.1093/ej/ueab033)

Please see Lines 341-355, 356-358(Table 4) in the revised manuscript.
