# Supplementary material for: Does the urban low-carbon transition promote the residents’ health consumption?
Source: PLoS One. 2025 Mar 21;20(3):e0319835. doi: 10.1371/journal.pone.0319835 (PMC11928064; doi:10.1371/journal.pone.0319835)
Supplement: Attachment — Submitted filename: Response_to_reviewers_auresp_2.docx [file pone.0319835.s005.docx]

**Responses to editor of PONE: regarding the paper** “Does the urban low-carbon transition promote the residents’ health consumption?” (Ref.: PONE-D-24-39039)

By

Pian Chen, Yanchi Chen

Dear Editor:

First of all, we would like to thank the reviewers for their serious and thorough work in refereeing this paper. All the reviewers’ suggestions have now been incorporated into the paper for revisions. A detailed description of the revisions undertaken is given below.

We think the reviewer’ queries and suggestions have been adequately addressed, and hope that the paper is now suitable for publication in PLOS ONE.

Thank you very much. We look forward to hearing from you.

Yours sincerely,

Corresponding author

Dr. Pian Chen

Institute of Food and Strategic Reserves

Nanjing University of Finance and Economics

Nanjing, 210023, China

**Point-by-point responses to the reviewers’ comments on the manuscript**

“Does the urban low-carbon transition promote the residents’ health consumption?” (Ref.: PONE-D-24-39039)

By

Pian Chen, Yanchi Chen

The authors would like to thank the Reviewers for their careful review of the manuscript and providing insightful comments and suggestions. The following responses have been prepared to address the reviewers’ comments. All the comments have been addressed in the revised manuscript (highlighted in red).

=============================================================

**Reviewer #2:**

***Comments 1.*** *The specific reasons for selecting only the CGSS data from 2018 and 2021 remain unclear. The CGSS data collection spans multiple periods, and the article states that the data from these two years were chosen because low-carbon pilot cities were implemented in 2010, 2012, and 2017. However, the low-carbon transition discussed in this paper uses carbon total factor productivity index as the explanatory variable, which does not conflict with the implementation of low-carbon city pilot policies. I suggest that the author select data from more years or provide a clearer explanation.*

**Response:** Thanks for your useful suggestions. We have provided a clearer explanation of the data used as suggested by the reviewers.

Please see Lines 294-305 in the revised manuscript.

***Comments 2.*** *Regarding the selection of the dependent variable, the article uses the number of participants in pension insurance and health insurance as proxy variables for health consumption. However, the author has not clearly explained the specific reasons for choosing pension insurance as a proxy for health consumption. I feel that the relationship between pension insurance and medical expenses is minimal, and this construction of the dependent variable is inappropriate.*

**Response:** Thanks for your useful suggestions. As suggested by the reviewers，we have modified the measure of population health consumption by applying the CGSS “whether or not commercial health insurance is purchased” to measure residents’ health consumption. We have also revised the other contents of the article.

Please see Lines 267-270, 311(Table 1), 314-323, 324(Table 2), 329-337, 341-342, 346(Table 3), 364 (Table 4), 391-393, 397(Table 5), 462(Table 6) in the revised manuscript.

***Comments 3.*** *In the benchmark regression results of Table 2, column (1) presents the findings using a random effects model. However, based on the research subjects and context of this study, employing a random effects model is not appropriate.*

**Response:** Thanks for your useful suggestions. We have removed the test associated with the random effects model as suggested by the reviewer.
