# Supplementary material for: Does the urban low-carbon transition promote the residents’ health consumption?
Source: PLoS One. 2025 Mar 21;20(3):e0319835. doi: 10.1371/journal.pone.0319835 (PMC11928064; doi:10.1371/journal.pone.0319835)
Supplement: Attachment — Submitted filename: Response to reviewer.docx [file pone.0319835.s006.docx]

**Responses to editor of PONE: regarding the paper** “Does the urban low-carbon transition promote the residents’ health consumption?” (Ref.: PONE-D-24-39039)

By

Pian Chen, Yanchi Chen

Dear Editor:

First of all, we would like to thank the reviewers for their serious and thorough work in refereeing this paper. The reviewer’ suggestions have now been incorporated into the paper for revisions. A detailed description of the revisions undertaken is given below.

We think the reviewer’ queries and suggestions have been adequately addressed, and hope that the paper is now suitable for publication in PLOS ONE.

Thank you very much. We look forward to hearing from you.

Yours sincerely,

Corresponding author

Dr. Pian Chen

Institute of Food and Strategic Reserves

Nanjing University of Finance and Economics

Nanjing, 210023, China

**Point-by-point responses to the reviewers’ comments on the manuscript**

“Does the urban low-carbon transition promote the residents’ health consumption?” (Ref.: PONE-D-24-39039)

By

Pian Chen, Yanchi Chen

The authors would like to thank the Reviewers for their careful review of the manuscript and providing insightful comments and suggestions. The following responses have been prepared to address the reviewers’ comments. All the comments have been addressed in the revised manuscript (highlighted in red).

=============================================================

**Reviewer #2:**

***Comments 1.*** *Regarding the measurement of health consumption indicators. In the revised manuscript, the article uses "whether to purchase commercial health insurance" as a measure of health consumption. It should be noted that why can commercial health insurance effectively measure individual health consumption? Further explanation is needed in the article. In addition, health consumption expenditure can usually be measured using health-related expenditure.*

**Response:** Thanks for your useful suggestions. We have added an explanation for the selection of “whether to purchase commercial health insurance” as a measure of residents’ health consumption. In addition, since the questionnaire of the China General Social Survey (CGSS) used in this paper does not contain specific content that directly reflects health-related expenditures, we are unable to use health expenditures as an indicator of health consumption. We hope for your understanding and thank you very much.

Please see Lines 267-282 in the revised manuscript.

***Comments 2***. *Regarding the selection of control variables. The selection of control variables in the article is relatively arbitrary, and the selection of control variables should strictly refer to relevant literature. The marital status, gender, household registration, family assets, and other variables of the head of the household can all affect the health consumption expenditure of the family, and these variables need to be controlled.*

**Response:** Thanks for your useful suggestions. We have added relevant control variables by reviewing existing research results and have revised the full text accordingly.

Please see Lines 300-305, 324(Table 1), 337(Table 2), 358(Table 3), 394(Table 4), 429(Table 5), 494(Table 6) in the revised manuscript.

***Comments 3.*** *Addressing endogeneity issues. The article uses instrumental variable method to solve the endogeneity problem of the model. Before selecting instrumental variables, it should be clearly explained what the sources of endogeneity in the model are? Additionally, how are instrumental variables measured and what are the basic descriptive results?*

**Response:** Thanks for your useful suggestions. In this paper, we have elaborated on the sources of endogeneity issues and explained how instrumental variables are measured. Meanwhile, we have further analyzed the statistical significance and economic significance of the regression results of the instrumental variables in depth to ensure the scientific validity and robustness of the findings.

Please see Lines 361-369,380-393 in the revised manuscript.

***Comments 4.*** *Details. For example, on page 16 of robustness test 4.2, the CGSS questionnaire "Do you participate in sports activities during leisure time" was used to measure health consumption, with a value of 3 for "how many times per week" and 3 for "how many times per month". The weekly assignment is the same as the monthly assignment, which is obviously not reasonable. It is recommended to verify.*

**Response:** Thanks for your useful suggestions. Upon verification, we have assigned a value of 2 to “how many times per month”.

Please see Lines 347-349 in the revised manuscript.
